# Voice-Based Remote Care Program for Vulnerable Older Adults in a Rural Community: Single-Arm Pilot Clinical Study

**DOI:** 10.2196/76653

**Published:** 2025-11-13

**Authors:** Geon Young Jang, Sunghwan Ji, Ji Yeon Baek, Eunju Lee, Seungryong Chong, Il-Young Jang

**Affiliations:** 1 Division of Geriatrics, Department of Internal Medicine, Asan Medical Center, University of Ulsan College of Medicine, 88, Olympic-ro 43-gil, Songpa-gu, Seoul, 05505, Republic of Korea, 82 230103308; 2 Department of Digital Health, Samsung Advanced Institute for Health Science & Technology, Sungkyunkwan University, Seoul, Republic of Korea; 3 AI Business Partnership Team, SK Telecom, Seoul, Republic of Korea; 4 PyeongChang Health Center and County Hospital, PyeongChang-gun, Gangwon-do, Republic of Korea

**Keywords:** caregiving, informal caregiver, remote care, smart speaker, telemedicine, digital health technology

## Abstract

**Background:**

Voice-based digital health technologies are highly feasible and acceptable tools for supporting older adults. However, their development has rarely focused on caregiving needs, and it is often poorly integrated with existing care services, thereby limiting their sustained effect.

**Objective:**

This study aimed to evaluate the feasibility and effectiveness of a comprehensive voice-based remote care program developed in partnership with a local public health center.

**Methods:**

A single-center, single-arm clinical study involving community-dwelling, socioeconomically vulnerable older adults was conducted using a Clinical Frailty Scale of 4‐5. Participants received a 6-month voice-based care program comprising smart speaker daily check-ins, an emergency response system, and artificial intelligence–driven well-being check calls. These components were integrated with the public health center for continuous monitoring. The primary outcome was caregiver burden, assessed using the Korean version of the Zarit Burden Interview. Secondary outcomes include depression (Patient Health Questionnaire-9), anxiety (Generalized Anxiety Disorder-7), and quality of life (Korean version of the Control, Autonomy, Self-realization, and Pleasure scale).

**Results:**

Among 100 enrolled participants, 96 (96%) completed the program. The caregiver burden slightly decreased from 17.1‐16.2 points (mean difference −1, 95% CI −2.17 to 0.24; *P*=.12). However, caregivers reported a significant reduction in their perception of being the sole support provider (*P*=.003). Among older adults, significant improvements were observed in depression (Patient Health Questionnaire-9; *P*<.001), anxiety (Generalized Anxiety Disorder-7; *P*=.008), and quality of life (Korean version of the Control, Autonomy, Self-Realization, and Pleasure scale; *P* =.048).. Program adherence was high, with participants engaging for a median of 184 (IQR 154‐203; 186/214, 87%) days.

**Conclusions:**

Whereas the voice-based remote care program did not significantly reduce the overall caregiver burden, it significantly reduced the perception of the caregivers as being the sole support system. Furthermore, it influenced the psychological well-being of older adults by reducing depression and anxiety and enhancing their quality of life. High adherence and engagement enhance the feasibility and acceptability of scalable digital health interventions for vulnerable older adults in rural settings.

## Introduction

Frailty is defined as a state of increased vulnerability resulting from age-related functional decline across multiple physiological systems, leading to decreased resilience to stress [1]. With global population aging, the number of older adults with frailty is steadily increasing [2]. In South Korea, the prevalence of frailty among individuals aged 65 years and older is approximately 23.1%. Given the rapid pace of population aging in South Korea, the absolute number of older adults with frailty is expected to continue increasing [3]. Older adults experiencing frailty require ongoing, long-term care, creating significant caregiver burden [4]. Informal caregivers, particularly family members or close friends, provide approximately 80% of long-term care in various countries [5]. These caregivers often face mental health challenges, including anxiety, depression, and burnout [6-8]. In addition, they face substantial financial strain, averaging US $7242 annually in the United States, US $10,800‐US 32,400 in South Korea, and US $6000-US $12,000 across Organisation for Economic Co-operation and Development countries [5,9,10]. Despite community support programs, workforce shortages and limited resources hinder continuous care [6,7,11-15], particularly in rural areas with inadequate support services [16].

Digital health technologies that enhance remote monitoring and home-based intervention are essential tools for easing caregiver burden [17,18]. However, digital technologies, including wearable devices, have innate limitations in terms of long-term adherence, particularly among older adults. Voice-based technologies, including smart speakers, demonstrate high feasibility and acceptability among older adults [19-23]. However, most digital health interventions focus on caregiver education or alleviating psychological distress [24-26], primarily addressing mental health, cognitive function, and social engagement [27-30]. In addition, most interventions are device-centric and lack integration with ongoing care services, limiting their ability to respond to evolving care needs [18].

Therefore, this study aims to evaluate the effectiveness of the program on the primary outcomes of caregiver burden and secondary outcomes, including well-being, depression, anxiety, and quality of life among older adults. To bridge this gap, we developed a comprehensive voice-based remote care program in partnership with a local public health center. This intervention integrates an artificial intelligence (AI)-powered speaker and an AI-driven interactive voice response (IVR) call system to provide daily check-ins and support for older adults with pre- to mild frailty in a rural South Korean community.

## Methods

### Trial Design and Study Participants

A single-arm clinical trial was conducted at PyeongChang Health Center and County Hospital (PCH), the principal public health facility in Pyeongchang County, South Korea. Participants were recruited from the Aging Study of the Pyeongchang Rural Area (ASPRA), a prospective cohort study targeting older adults in the region. The specific protocol for the ASPRA cohort has previously been published [31]. Briefly, the ASPRA inclusion criteria required participants to be ≥65 years old, enrolled in the National Healthcare Service, and able to walk with or without aid. In addition, they needed to live within the community (noninstitutionalized) and be capable of providing informed consent personally or through a legal proxy. Individuals living in nursing homes, hospitalized, or receiving equivalent care at home were excluded. All study participants underwent an annual Comprehensive Geriatric Assessment.

Overall, 1955 older adults were screened from the ASPRA database. The selection was based primarily on frailty and socioeconomic vulnerability. Eligible participants were ≥65, residing within the community, with a Clinical Frailty Scale (CFS) of 4‐5, and living alone or with low income. Individuals with significant cognitive impairments limiting the use of smart speakers, those with severe hearing loss unable to manage incoming calls, and those with <1-year life expectancy based on patient-reported terminal conditions (metastatic cancer or other end-stage diseases) were excluded.

Since this study involved a novel intervention with limited prior research, formal sample size calculations based on statistical power were not applicable. A target sample size of 100 participants was determined through consultation with local health care stakeholders, considering feasibility and regional context.

### Intervention

**Figure 1.**
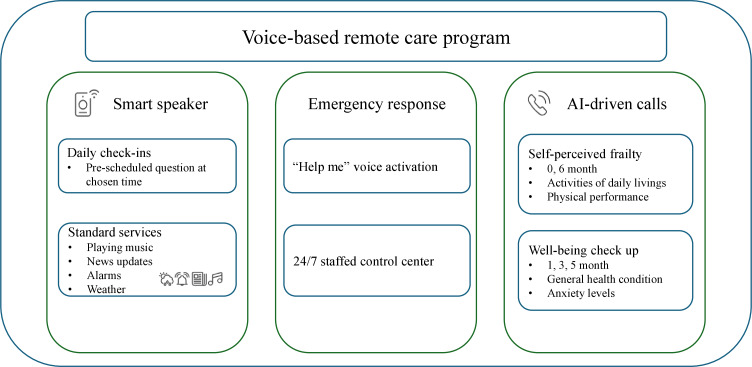
Conceptual framework of a voice-based remote care program featuring 3 key components: smart speaker, emergency response, and AI-driven calls. AI: artificial intelligence.

This voice-based remote care program is a structured intervention designed to provide continuous in-home support for older adults using integrated smart technologies. Participants were enrolled in a 6-month program operated by PCH, which delivered services through a smart speaker (SKT NUGU by SK Telecom, Seoul, Korea) and an IVR system. The intervention comprised 3 key components: smart speaker-based daily check-ins, an emergency response system, and AI-driven well-being check calls (care calls), Figure 1.

Smart speaker-based daily check-ins allowed participants to receive simple, prescheduled questions at their preferred time. These questions consisted of general wellness inquiries with health behavior recommendations (Multimedia Appendix 1). The smart speaker provided immediate responses tailored to inputs, including interactive health guidance and retrieval of relevant health information. When participants requested immediate assistance, the system facilitated direct phone calls to their caregivers or PCH staff. The system automatically logged the participant’s response status (responded or did not respond). If no response was detected within a specified period (default: 3 days, adjustable based on participant preference), the system flagged the case for review by PCH staff during regular working days. Staff then conducted follow-up phone calls to participants or their caregivers to confirm their status. In addition, the smart speakers provided standard services, including music playback, news updates, alarm settings, and weather forecasts.

The emergency response system, also managed through a smart speaker, enabled participants to request immediate assistance during falls or other home emergencies. Participants could activate the smart speaker by saying “Help me,” to connect directly to a 24/7 staffed control center managed by a private security company. Control center staff assessed the situation in real time via the smart speaker or through a follow-up phone call and, if necessary, promptly dispatched emergency services for immediate intervention. All activations, including details of communications between participants and control center staff, were recorded for monitoring and quality assurance purposes.

The AI-driven well-being check calls used an IVR system to conduct periodic well-being assessments throughout the 6-month intervention period. The IVR system incorporated AI technology (NUGU Interactive technology by SK Telecom), which includes speech recognition, natural language understanding, and dialog management. During this period, the system made 5 scheduled calls to each participant. At baseline and 6 months, comprehensive self-perceived frailty assessments were conducted using a branching questioning algorithm that included Activities of Daily Living (ADL), Instrumental Activities of Daily Living (IADL), and physical performance measures. At 1, 3, and 5 months, brief health status and anxiety assessments were administered. The AI system automatically interpreted participant responses, classified general health status as positive or negative, determined self-perceived frailty scores, and identified newly developed disabilities in ADL or IADL. PCH staff monitored these categorized responses monthly. When participants showed signs of deterioration, they were advised to visit PCH or other health care facilities.

At initial setup, PCH staff visited participant homes to install and configure smart speakers and Wi-Fi systems, providing user training. Technical support was available throughout the intervention period, with participants able to contact PCH for basic troubleshooting or specialized technical assistance for complex issues.

### Measurements

Participants underwent a comprehensive geriatric assessment. Demographic data collected included age, sex, height, weight, medical aid status, living arrangement (living alone or not), caregiver relationship, and smartphone usage. Comorbidities and the number of regularly prescribed medications were also recorded. Physical performance was assessed using the short physical performance battery, which ranges from 0‐12, along with a measurement of handgrip strength [32]. Disability status was defined as the presence of 1 or more impairments in any of the 7 ADL items or 10 IADL items [33]. Cognitive function was evaluated using the Mini-Cog test, with cognitive dysfunction defined as a score of ≤3 [34]. Frailty was assessed using the Korean version of the CFS and a 45-item Frailty Index (Multimedia Appendix 2) [35]. All assessments were conducted in person by trained nurses at PCH.

### Outcomes

The primary outcome was caregiver burden, measured using the Korean version of the Zarit Burden Interview (ZBI-K) [36], administered to caregivers in person at baseline and the end of the 6-month intervention. The ZBI-K comprised 22 items, each rated on a 5-point Likert scale (0=never to 4=always), yielding an overall score range of 0‐88 [37]. Higher ZBI-K scores indicated a greater level of caregiver burden and were categorized as follows: 0‐20 (little or no burden), 21‐40 (mild to moderate burden), 41‐60 (moderate to severe burden), and 61‐88 (severe burden) [38,39]. Multimedia Appendix 3 provides the complete ZBI-K questionnaire.

Secondary outcomes included changes in psychological well-being and quality of life among older adult participants, assessed in person at PCH at baseline and 6 months. Depressive symptoms were assessed using the Patient Health Questionnaire-9 (PHQ-9), a 9-item instrument with an overall score range of 0‐27 [40,41]. Anxiety levels were measured using the Generalized Anxiety Disorder-7 (GAD-7), a 7-item scale ranging from 0‐21 [42,43]. Quality of life was evaluated using the Korean version of the Control, Autonomy, Self-Realization, and Pleasure Scale (K-CASP-19), which has a score range of 0‐57 [44,45] and the EuroQol-5 Dimension [46,47].

In addition, smart speaker usage patterns were monitored throughout the intervention period. Data collected included the number of days the smart speaker was used, total usage frequency, and average daily interactions. The activation of the emergency response system and engagement with the AI telephone service (care call response rate) were also recorded to assess feasibility and participant compliance.

### Statistical Methods

Baseline characteristics were summarized using means (SD) values for continuous variables and frequencies with percentages for categorical variables. To evaluate changes in primary and secondary outcomes over time, a linear mixed-effect model was used, specifying time as a fixed effect and participant ID as a random effect. Estimated marginal means were calculated for each time point, and pairwise comparisons were performed to assess mean differences between baseline and follow-up assessments. CI values for mean changes were calculated, and effect sizes were estimated using Cohen *d*. For individual ZBI-K items, pre–post differences were evaluated using the Wilcoxon signed-rank test. All statistical analyses were performed using R version 4.4.1 (R Core Team), with a significance level set at α=.05.

### Ethical Considerations

The trial was registered with the Clinical Research Information Service of the Republic of Korea (identifier: KCT0009015) and conducted in accordance with the Declaration of Helsinki. Ethical approval was obtained from the Institutional Review Board of Asan Medical Center (institutional review board approval number 2023-0587). All participants provided written informed consent prior to enrollment. Participant confidentiality was strictly maintained, and all data were anonymized before analysis. No monetary compensation was provided for participation.

## Results

### Participants

**Figure 2.**
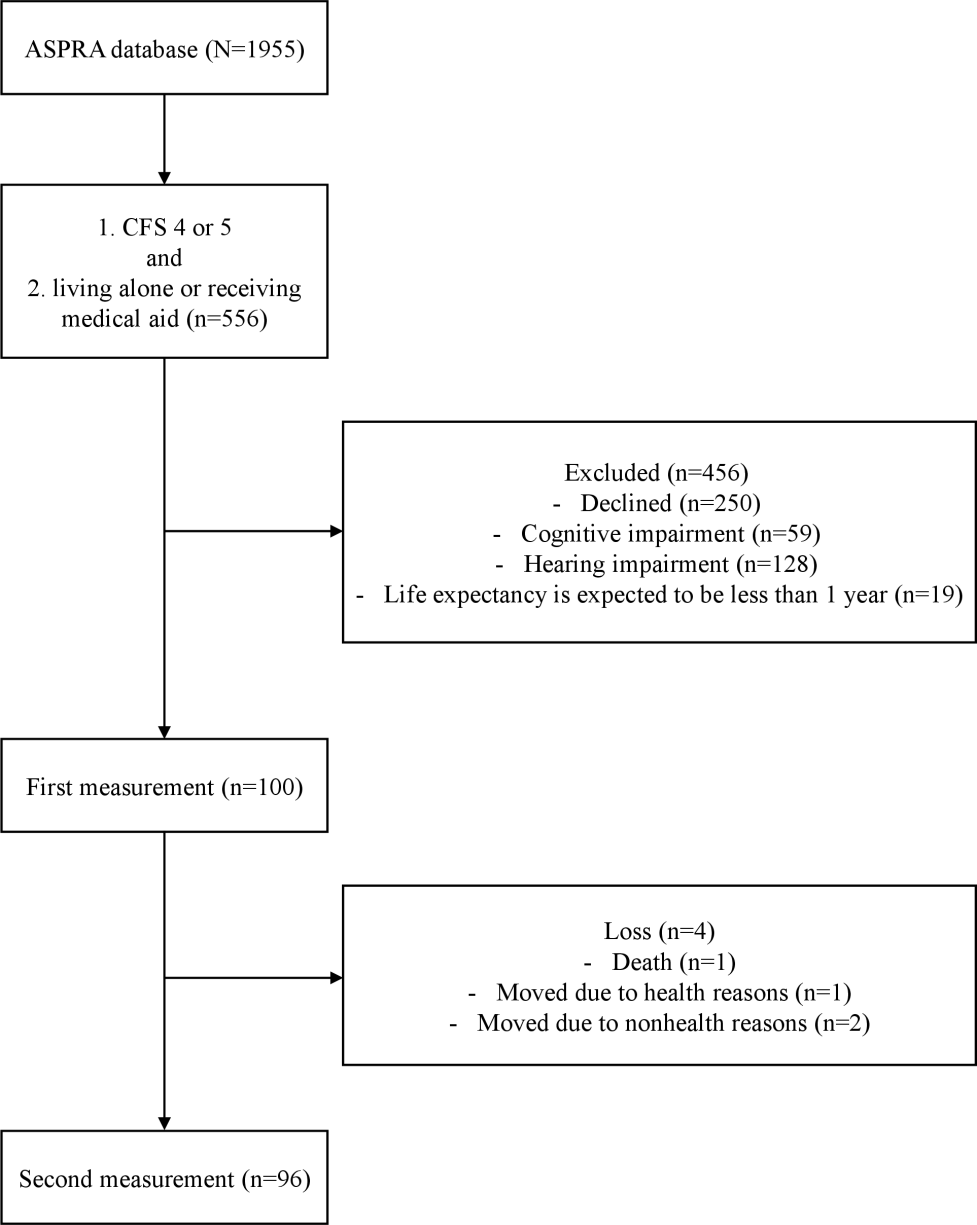
Flowchart of participant selection and follow-up. ASPRA: Aging Study of the Pyeongchang Rural Area; CFS: Clinical Frailty Scale.

Between June 1 and December 31, 2023, 556 participants were identified from the ASPRA database based on the eligibility criteria: a CFS score of 4 or 5 and either living alone or receiving medical aid. However, 456 individuals were excluded owing to refusal to participate (n=250), cognitive impairment (n=59), hearing impairment (n=128), or a life expectancy of <1 year (n=19). Consequently, 100 participants were enrolled in the study (Figure 2). During the study period, 4 participants withdrew from the intervention: 1 owing to death, 2 owing to relocation for non–health-related reasons, and 1 relocated for health-related reasons. Consequently, 96 participants completed the second measurement. Table 1 shows the baseline characteristics of the participants. The majority were female (84/100, 84%), with a mean age of 76.5 (SD 6.1) years. A significant proportion of participants (66/100, 66%) lived alone and most were capable of using smartphones (80/100, 80%).

Regarding caregiving support, most participants received care from their immediate family members. Among male participants, the primary caregivers were most often their spouses (10/16, 62.5%). In contrast, female participants were predominantly cared for by their children (58/84, 70.2%). A smaller proportion of the participants received care from their daughters-in-law (5/84, 6%), siblings (3/84, 3.6%), or other caregivers (2/84, 2.4%).

**Table 1.** Baseline characteristics of the study groups.

Characteristics	Male (n=16)	Female (n=84)
Age (years), mean (SD)	75.69 (6.83)	76.70 (5.88)
Medical aid, n (%)	3 (18.8)	9 (10.7)
Living alone, n (%)	6 (37.5)	59 (70.2)
Smartphone usage, n (%)	14 (87.5)	66 (78.6)
Caregiver, n (%)		
Spouses	10 (62.5)	16 (19)
Children	6 (37.5)	58 (70.2)
Daughters-in-law	0 (0)	5 (6)
Siblings	0 (0)	3 (3.6)
Others	0 (0)	2 (2.4)
BMI (kg/m^2^), mean (SD)	25.66 (2.61)	26.02 (4.24)
Skeletal muscle index (kg/m^2^), mean (SD)	7.46 (0.70)	6.12 (0.88)
Gait speed (m/s), mean (SD)	0.78 (0.18)	0.81 (0.28)
SPPB^a^ (score), mean (SD)	8.80 (1.61)	8.70 (2.38)
Grip strength (kg), mean (SD)	30.49 (8.49)	21.13 (5.15)
Mini-Cog≤3, n (%)	9 (56.2)	50 (59.5)
No. of chronic conditions, mean (SD)	2.25 (1.48)	2.46 (1.43)
No. of medication, mean (SD)	4.25 (2.62)	3.56 (2.39)
Fall in the last year, n (%)	4 (25)	36 (42.9)
ADL^b^ disability, n (%)	2 (12.5)	44 (52.4)
IADL^c^ disability, n (%)	3 (18.8)	42 (50)
CFS^d^, mean (SD)	3.44 (0.63)	3.57 (0.72)
Frailty index, mean (SD)	0.19 (0.09)	0.25 (0.12)

a SPPB: short physical performance battery.

b ADL: activities of daily living.

c IADL: instrumental activities of daily living.

d CFS: Clinical Frailty Scale.

### Primary Outcome

Caregiver burden, assessed using the Korean version of the ZBI-K, showed a slight, nonsignificant reduction more than the 6-month intervention period. The mean score decreased from 17.1 at baseline to 16.2 at follow-up, with a mean difference of −1 (95% CI −2.17 to 0.24; *P*=.12). When individual ZBI-K items were analyzed, most showed minimal or nonsignificant changes. However, item 14—which assessed the perception of the caregivers as being the sole source of support— demonstrated a significant reduction from baseline to follow-up (mean change −0.43, Cohen *d*=−0.34; *P*=.003). Multimedia Appendix 4 presents details of other item-level analyses.

### Secondary Outcomes

Regarding psychological function, anxiety levels, as measured by the GAD-7 scale, decreased significantly from a mean score of 2.97 to 2.18 (mean change −0.79, 95% CI −1.37 to −0.81; *P*=.008). Similarly, depression, assessed using the PHQ-9, showed a significant reduction from a mean score of 5.68 to 4.02 (mean change −1.66, 95% CI −2.41 to −0.90; *P*<.001).

Quality of life, evaluated via the K-CASP-19, significantly improved, with scores increasing from 27.3‐28.8 (mean change 1.53, 95% CI 0.01‐3.05; *P*=.048). However, the EuroQol-5 Dimension score exhibited only a minimal increase from 0.77 to 0.78, which was not statistically significant (mean change 0.01, 95% CI −0.01 to 0.03; *P*=.46). A summary of the primary and secondary outcomes is presented in Table 2.

**Table 2.** Primary and secondary outcomes.

Outcomes	Values, mean (SD)		Change		
	Baseline	Follow-up	Mean change (95% CI)	Cohen *d*	*P* value^a^
Primary outcome					
ZBI-K^b^	17.1 (7.9)	16.2 (8.9)	−1.0 (−2.17 to 0.24)	−0.15	.12
Secondary outcomes					
GAD-7^c^	2.97 (3.49)	2.18 (2.87)	−0.79 (−1.37 to −0.81)	−0.23	.008
K-CASP-19^d^	27.3 (7.6)	28.8 (8.0)	1.53 (0.01 to 3.05)	0.21	.048
EQ-5D^e^	0.77 (0.12)	0.78 (0.01)	0.01 (−0.01 to 0.03)	0.08	.46
PHQ-9^f^	5.68 (4.86)	4.02 (3.73)	−1.66 (−2.41 to −0.90)	−0.37	<.001

a Linear mixed-effects model.

b ZBI-K: Korean version of the Zarit Burden Interview.

c GAD-7: Generalized Anxiety Disorder-7.

d K-CASP-19: Korean version of the Control, Autonomy, Self-realization, and Pleasure scale–19 items.

e EQ-5D: EuroQol 5-dimension questionnaire.

f PHQ-9: Patient Health Questionnaire-9.

### Technology Usage and Compliance

Overall, the participants exhibited high usage rates of the smart speaker device. Multimedia Appendix 5 illustrates that the participants used the smart speaker for a median of 184 (IQR 154‐203.25) days out of the total 214 days, representing approximately 87% (186/214) of the intervention period. Total usage frequency varied across participants, with a median of 508 interactions (IQR 374.75‐729.25). The lowest usage was 7, whereas the highest reached 2136 interactions, highlighting the diverse usage patterns. On average, daily usage was 2.38 interactions (IQR 1.75‐3.41), with individual rates ranging from 0.03‐9.98 interactions per day.

Multimedia Appendix 6 shows that the AI-driven well-being check calls demonstrated high response rates throughout the study. At baseline (0 months), all participants (100/100, 100%) responded to the care call. This response rate remained unchanged for 1 month (99/99, 100%) and 3 months (98/98, 100%). However, in the 5-month assessment, the response rate decreased to 91.8% (90/97) among the 97 remaining participants. Reasons for nonresponse at the 5-month mark included connection failures (n=5), inability to verify identity (n=3), and refusal to engage in conversation (n=1). By the final 6-month assessment, the response rate returned to 100% among the 96 participants who completed the study.

The emergency response system, specifically the “Help me” function, was activated 3 times during the study period. However, each activation was determined to be a false alarm caused by the misinterpretation of voice commands rather than actual emergency situations. No genuine emergency events requiring intervention were recorded throughout the study period.

## Discussion

### Principal Findings

This pilot study evaluated a voice-based remote care system, integrating an AI speaker and IVR call system to support vulnerable older adults in rural South Korea, focusing on caregiver burden, psychological well-being, and intervention feasibility. Although the primary outcome—caregiver burden, measured using the ZBI-K—did not show a statistically significant improvement, the total score showed slight improvement. In addition, item-level analysis revealed a significant reduction in the perception of caregivers as the sole provider of support. Older adults showed significant improvements in depression, anxiety, and quality of life. High engagement with the smart speaker, along with near-complete response rates to AI-driven check-in calls, supports the feasibility and acceptability of the intervention.

The mean ZBI-K score showed a slight decrease from 17.1 at baseline to 16.2 at follow-up (*P*=.12). This minimal change may be partly attributed to the low baseline burden among caregivers, as a score of 17.1 typically indicates low burden [38,39]. Since our participants were community-dwelling older adults, primarily socioeconomically vulnerable and ranging from prefrail to mildly frail, the caregiver burden was relatively low compared to those of caregivers of individuals with multiple chronic conditions (mean ZBI-K 38.27, SD 14.58) [48] or dementia (mean ZBI-K 51.09, SD 17.95) [49].

Nevertheless, ZBI-K item 14 (“Do you feel that your relative seems to expect you to take care of them as if you were the only one they could depend on?”) showed significant improvement with a mean reduction of 0.43 points (Cohen *d*=−0.34; *P*=.003). It specifically measures the caregiver’s perception of being the sole provider of support. Previous qualitative studies have similarly suggested that voice-based devices provide caregivers with psychological reassurance, emotional support, scheduling assistance, and emergency response [50-52]. Direct comparisons with these studies are challenging due to differences in research settings, such as dementia status, caregiver type, and living arrangements. Despite these limitations, our findings provide quantitative support for the psychological reassurance and reduced caregiving responsibility indicated by earlier qualitative research.

This intervention had significant effects on secondary outcomes assessed among older adult participants. Notable improvements were observed in depression (PHQ-9 scores decreased from 5.68 to 4.02, mean change −1.66; *P*<.001), anxiety (GAD-7 scores decreased from 2.97 to 2.18, mean change −0.79*; P*=.008), and quality of life (K-CASP-19 scores increased from 27.3 to 28.8, mean change 1.53; *P*=.048). These findings align with previous qualitative studies, which suggested companionship, enhanced social connectedness, and personalization through user experiences as potential mechanisms underlying improvements in loneliness and depression [27-29]. While our study used standardized assessment tools and quantitatively demonstrated psychological improvements, it did not directly investigate these mechanisms. Future research could further explore these proposed hypotheses.

This intervention demonstrated 3 key strengths that contributed to overall effectiveness, high adaptability, and sustainability of this program. First, it is a voice-based system using an AI-powered speaker and IVR calls, providing a more user-friendly interface for older adults compared to smartphones or wearable devices, which often present usability challenges [53]. Second, the program integrates 3 core components—smart speaker-based daily check-ins, emergency response system, and AI-driven well-being check calls—into a single, seamless system that connects directly with the local public health infrastructure. Unlike previous interventions, which typically focused on a single component [20-22,27-30,50-52,54,55], this multi-layered design offers more comprehensive support and responsive care. Third, the intervention was designed to reduce dependence on human resources, addressing a key limitation of traditional telecare systems such as videoconferencing or telephone support, which usually require active involvement from health care professionals, thereby limiting scalability [17,56].

This study has some limitations. First, the absence of a control group limits our ability to establish causality, as improvements in secondary outcomes may have been influenced by external factors. However, to mitigate selection bias, we included all eligible participants from the ASPRA cohort database who met our inclusion criteria without additional selection processes. Second, the intervention primarily focused on care recipients rather than caregivers, which may explain the lack of significant improvement in overall caregiver burden. Third, the focus on rural older adults is a notable strength of this study, addressing a population typically underrepresented in digital health research; however, these findings may not be directly applicable to urban settings or different cultural settings. Fourth, our study was limited to informal caregivers. Formal caregivers differ from informal caregivers in their expectations toward voice-based technology, particularly regarding professional accuracy and efficiency versus emotional support.

Therefore, future research should implement randomized controlled trial designs to establish causality. In addition, incorporating more diverse populations and expanding the intervention to support caregivers alongside care recipients may offer a more comprehensive approach to reducing caregiver burden. A mixed-methods approach, combining quantitative assessments with qualitative feedback from caregivers, could also provide valuable insights into psychological improvements and practical impacts of the program.

### Conclusions

Voice-based remote care program using smart speakers and AI-driven telephone services for vulnerable older adults in rural areas did not significantly reduce the overall caregiver burden. However, it reduced the perception of being the sole support provider. For participants, the intervention improves psychological well-being, significantly reducing depression and anxiety and enhancing quality of life. High engagement and adherence confirm the feasibility and acceptability of this intervention among older adults in rural settings. These findings highlight the potential of smart speaker-based remote care as a scalable supplement to traditional services in rural areas, improving psychological well-being and achieving high acceptance among vulnerable older adults .

## Supplementary material

10.2196/76653 Multimedia Appendix 1 Two-week examples of questions from “the smart speaker-based daily check-ins”.

10.2196/76653 Multimedia Appendix 2 The 45 items of the frailty index.

10.2196/76653 Multimedia Appendix 3 Zarit Burden interview.

10.2196/76653 Multimedia Appendix 4 Changes in individual Korean version of the Zarit Burden Interview items from baseline to 6-month follow-up.

10.2196/76653 Multimedia Appendix 5 Smart speaker usage patterns during the 6-month intervention period.

10.2196/76653 Multimedia Appendix 6 Care call response rates throughout the intervention period.

## Abbreviations

ADL: activities of daily living
AI: artificial intelligence
ASPRA: Aging Study of Pyeongchang Rural Area
CFS: Clinical Frailty Scale
GAD-7: Generalized Anxiety Disorder-7
IADL: instrumental activities of daily living
IVR: interactive voice response
K-CASP-19: Korean version of the Control, Autonomy, Self-Realization, and Pleasure scale
PCH: Pyeongchang County Health Center and County Hospital
PHQ-9: Patient Health Questionnaire-9
ZBI-K: Korean version of Zarit Burden Interview
