# Supplementary material for: Voice-Based Remote Care Program for Vulnerable Older Adults in a Rural Community: Single-Arm Pilot Clinical Study
Source: JMIR Aging. 2025 Nov 13;8:e76653. doi: 10.2196/76653 (PMC12616100; doi:10.2196/76653)
Supplement: Multimedia Appendix 1 [file aging-v8-e76653-s001.docx]

**Table S1.**

| Week | Day | Question |
| --- | --- | --- |
| 1 | Monday | Did you brush your teeth after meals today? |
| 1 | Tuesday | Did you take your daily medication today? |
| 1 | Wednesday | Did you exercise for > 30 min today? |
| 1 | Thursday | Did you eat your meals properly today? |
| 1 | Friday | Did you feel sad or depressed today? |
| 1 | Saturday | Did you meet and talk with family, friends, or neighbors today? |
| 1 | Sunday | Did you experience any falls during the past week? |
| 2 | Monday | How many cups of water did you drink today? |
| 2 | Tuesday | Did you sleep well today? |
| 2 | Wednesday | Have you tried the dementia-prevention cognitive service built into the smart speaker? |
| 2 | Thursday | Are you familiar with frailty? |
| 2 | Friday | Do you know about sarcopenia (muscle loss)? |
| 2 | Saturday | Do you have bowel movements fewer than three times a week? |
| 2 | Sunday | Do you digest food well after meals? |
