# Supplementary material for: Voice-Based Remote Care Program for Vulnerable Older Adults in a Rural Community: Single-Arm Pilot Clinical Study
Source: JMIR Aging. 2025 Nov 13;8:e76653. doi: 10.2196/76653 (PMC12616100; doi:10.2196/76653)
Supplement: Multimedia Appendix 2 [file aging-v8-e76653-s002.docx]

**Table S1.**

| Dressing | Low muscle mass |
| --- | --- |
| Face washing | Low grip strength |
| Bathing | Chair stand test |
| Eating | Balance test |
| Mobility | Gait speed test |
| Using the restroom | Mini-Cog |
| Bowel and bladder control | Patient Health Questionnaire-9 |
| Dressing wounds | Mini Nutritional Assessment-Short Form |
| Household chores | Low physical activity |
| Meal preparation | Polypharmacy |
| Laundry | History of falls in 1 year |
| Short-distance outings | History of stroke |
| Using transportation | Hypertension |
| Shopping | Diabetes mellitus |
| Managing finances | Dyslipidemia |
| Using the telephone | Thyroid disease |
| Taking medication | Coronary artery disease |
| Physical fitness | Asthma |
| Self-management | Atopy |
| Daily activities (work, study, etc.) | Arthritis |
| Pain/discomfort | Chronic kidney disease |
| Anxiety/depression | Liver disease |
|  | Pulmonary tuberculosis |
