# Supplementary material for: Voice-Based Remote Care Program for Vulnerable Older Adults in a Rural Community: Single-Arm Pilot Clinical Study
Source: JMIR Aging. 2025 Nov 13;8:e76653. doi: 10.2196/76653 (PMC12616100; doi:10.2196/76653)
Supplement: Multimedia Appendix 3 [file aging-v8-e76653-s003.docx]

**Table S1.**

| No. | Question | Score (circle one) |
| --- | --- | --- |
| 1 | Do you feel that your relative asks for more help than they need? | 0   1   2   3   4 |
| 2 | Do you feel that because of the time you spend with your relatives, you do not have enough time for yourself? | 0   1   2   3   4 |
| 3 | Do you feel stressed between caring for your relative and trying to meet other responsibilities for your family or work? | 0   1   2   3   4 |
| 4 | Do you feel embarrassed over the behavior of your relatives? | 0   1   2   3   4 |
| 5 | Do you feel angry when you are around your relatives? | 0   1   2   3   4 |
| 6 | Do you feel that your relative currently affects your relationships with other family members or friends negatively? | 0   1   2   3   4 |
| 7 | Are you afraid of what the future holds for your relative? | 0   1   2   3   4 |
| 8 | Do you feel your relative is dependent on you? | 0   1   2   3   4 |
| 9 | Do you feel strained when you are around your relatives? | 0   1   2   3   4 |
| 10 | Do you feel your health has suffered because of your involvement with your relative? | 0   1   2   3   4 |
| 11 | Do you feel that you do not have as much privacy as you would like because of your relatives? | 0   1   2   3   4 |
| 12 | Do you feel that your social life has suffered because you are caring for your relative? | 0   1   2   3   4 |
| 13 | Do you feel uncomfortable about having friends over because of your relatives? | 0   1   2   3   4 |
| 14 | Do you feel that your relative seems to expect you to take care of them as if you were the only one they could depend on? | 0   1   2   3   4 |
| 15 | Do you feel that you do not have enough money to take care of your relative in addition to the rest of your expenses? | 0   1   2   3   4 |
| 16 | Do you feel that you will be unable to take care of your relative much longer? | 0   1   2   3   4 |
| 17 | Do you feel you have lost control of your life since the illness of your relative? | 0   1   2   3   4 |
| 18 | Do you wish you could leave the care of your relative to someone else? | 0   1   2   3   4 |
| 19 | Do you feel uncertain about what to do about your relative? | 0   1   2   3   4 |
| 20 | Do you feel you should be doing more for your relative? | 0   1   2   3   4 |
| 21 | Do you feel you could do a better job in caring for your relative? | 0   1   2   3   4 |
| 22 | Overall, how burdened do you feel in caring for your relative? | 0   1   2   3   4 |

Responses were scored based on the frequency of caregivers’ feelings as follows:

- 0: Never
- 1: Rarely
- 2: Sometimes
- 3: Quite frequently
- 4: Nearly always

Interpretation of Total Scores

| Total Score | Interpretation |
| --- | --- |
| 0 - 20 | Little or no burden |
| 21 - 40 | Mild to moderate burden |
| 41 - 60 | Moderate to severe burden |
| 61 - 88 | Severe burden |
