# Supplementary material for: Voice-Based Remote Care Program for Vulnerable Older Adults in a Rural Community: Single-Arm Pilot Clinical Study
Source: JMIR Aging. 2025 Nov 13;8:e76653. doi: 10.2196/76653 (PMC12616100; doi:10.2196/76653)
Supplement: Multimedia Appendix 4 [file aging-v8-e76653-s004.docx]

**Table S1.**

| ZBI-K Item | Baseline | Follow-up | Mean Difference | *P*-value | Cohen *d* |
| --- | --- | --- | --- | --- | --- |
| Item 1 | 0.52 | 0.36 | -0.16 | .113^b^ | -0.17 |
| Item 2 | 0.32 | 0.32 | 0.00 | .914 ^b^ | 0.00 |
| Item 3 | 0.52 | 0.51 | -0.01 | .958 ^b^ | -0.01 |
| Item 4 | 0.11 | 0.15 | 0.04 | .468 ^b^ | 0.09 |
| Item 5 | 0.41 | 0.45 | 0.04 | .568 ^b^ | 0.05 |
| Item 6 | 0.04 | 0.09 | 0.05 | .281 ^b^ | 0.14 |
| Item 7 | 2.85 | 2.83 | -0.02 | .849 ^b^ | -0.01 |
| Item 8 | 1.66 | 1.56 | -0.10 | .627 ^b^ | -0.08 |
| Item 9 | 0.37 | 0.44 | 0.07 | .364 ^b^ | 0.09 |
| Item 10 | 0.11 | 0.11 | 0.00 | .916 ^b^ | 0.00 |
| Item 11 | 0.36 | 0.35 | -0.01 | .974 ^b^ | -0.01 |
| Item 12 | 0.21 | 0.31 | 0.10 | .269 ^b^ | 0.14 |
| Item 13 | 0.07 | 0.10 | 0.03 | .546 ^b^ | 0.07 |
| Item 14 | 1.03 | 0.60 | -0.43 | .003 ^b^ | -0.34 |
| Item 15 | 0.76 | 0.71 | -0.05 | .748 ^b^ | -0.04 |
| Item 16 | 0.15 | 0.22 | 0.07 | .187 ^b^ | 0.10 |
| Item 17 | 0.11 | 0.16 | 0.05 | .296 ^b^ | 0.10 |
| Item 18 | 0.14 | 0.14 | 0.00 | .872 ^b^ | 0.00 |
| Item 19 | 0.46 | 0.44 | -0.02 | .865 ^b^ | -0.02 |
| Item 20 | 2.77 | 2.71 | -0.06 | .709 ^b^ | -0.04 |
| Item 21 | 3.06 | 3.11 | 0.05 | .696 ^b^ | 0.04 |
| Item 22 | 0.78 | 0.93 | 0.15 | .139 ^b^ | 0.13 |
| Total Score | 16.81 | 16.64 | -0.17 | .849 ^c^ | -0.02 |

^a^Korean version of the Zarit Burden Interview

^b^Wilcoxon signed-rank test.

^c^Linear mixed-effects model.
