# Supplementary material for: Voice-Based Remote Care Program for Vulnerable Older Adults in a Rural Community: Single-Arm Pilot Clinical Study
Source: JMIR Aging. 2025 Nov 13;8:e76653. doi: 10.2196/76653 (PMC12616100; doi:10.2196/76653)
Supplement: Multimedia Appendix 5 [file aging-v8-e76653-s005.docx]

**Table S1**.

| Metric | Median | IQR | Maximum | Minimum |
| --- | --- | --- | --- | --- |
| Total Days Used^a^ | 184 | 154-203.25 | 214 | 4 |
| Total Usage^b^ | 508 | 374.75-729.25 | 2136 | 7 |
| Average Daily Usage^c^ | 2.38 | 1.75-3.41 | 9.98 | 0.03 |

^a^Total Days Used represents the number of days the smart speaker was active during the study period (maximum possible: 214 days).

^b^Total usage indicates the number of distinct interactions with the device.

^c^Average Daily Usage was calculated as the total interactions divided by the number of days used.
