# Supplementary material for: Voice-Based Remote Care Program for Vulnerable Older Adults in a Rural Community: Single-Arm Pilot Clinical Study
Source: JMIR Aging. 2025 Nov 13;8:e76653. doi: 10.2196/76653 (PMC12616100; doi:10.2196/76653)
Supplement: Multimedia Appendix 6 [file aging-v8-e76653-s006.docx]

**Table S1.**

| Month | Participants (n) | Response Rate (%) | Reasons for Non-response |
| --- | --- | --- | --- |
| 0 | 100 | 100 | - |
| 1 | 99 | 100 | - |
| 3 | 98 | 100 | - |
| 5 | 97 | 91.8 | Connection failure (5),  Identity verification failure (3),  Conversation refusal (1) |
| 6 | 96 | 100 | - |
